# A predictive algorithm for the optimal daily dosage of thiamazole to control cats with hyperthyroidism

**DOI:** 10.1093/jvimsj/aalag009

**Published:** 2026-02-03

**Authors:** Pak-Kan Tang, Nicola Lötter, Rebecca F Geddes, Rosanne E Jepson, Yu-Mei Chang, Harriet Syme, Jonathan Elliott

**Affiliations:** Department of Comparative Biomedical Sciences, Royal Veterinary College, University of London, London NW1 0TU, United Kingdom; Department of Comparative Biomedical Sciences, Royal Veterinary College, University of London, London NW1 0TU, United Kingdom; Department of Clinical Science and Services, Royal Veterinary College, University of London, London AL9 7TA, United Kingdom; Department of Clinical Science and Services, Royal Veterinary College, University of London, London AL9 7TA, United Kingdom; Department of Comparative Biomedical Sciences, Royal Veterinary College, University of London, London NW1 0TU, United Kingdom; Department of Clinical Science and Services, Royal Veterinary College, University of London, London AL9 7TA, United Kingdom; Department of Comparative Biomedical Sciences, Royal Veterinary College, University of London, London NW1 0TU, United Kingdom

**Keywords:** dose optimization, thiamazole, methimazole, predictive model, probability, validation

## Abstract

**Background:**

Hyperthyroidism is the most common endocrinopathy in cats and is frequently managed using anti-thyroid medication.

**Hypothesis/Objectives:**

To develop and validate an algorithm to predict the optimal starting daily dose of thiamazole required to control hyperthyroidism in cats.

**Animals:**

One hundred eighty-eight client-owned cats with hyperthyroidism for algorithm development (2011-2021) and 45 hyperthyroid cats to validate the algorithm (2022-2024).

**Methods:**

Retrospective case-control study. Cats with hyperthyroidism controlled medically using thiamazole within a year since diagnosis were enrolled. Controlled dose of thiamazole was categorized into “≤5 mg” or “>5 mg.” Binary logistic regression was performed to explore predictors associated with thiamazole dose. The performance of the final multivariable model in prediction was assessed by receiver operating characteristic (ROC) curve analysis. A cohort of cats subsequently diagnosed with hyperthyroidism and managed chronically with thiamazole were used to test algorithm performance.

**Results:**

At hyperthyroidism diagnosis, baseline plasma total thyroxine (TT4); (odds ratio [OR] 1.29 [95% CI, 1.19-1.42] per 10 nmol/L; *P* < .001) and creatinine concentrations (OR 0.83 [95% CI, 0.7-0.96] per 0.1 mg/dL; *P* = .02) were independent predictors for higher thiamazole dose (>5 mg). The area under the ROC curve was 0.92 (95% CI, 0.88-0.96). In the test cohort, 26 cats controlled on ≤ 5 mg and 19 required >5 mg thiamazole. The predictive model had overall accuracy of 91.1%, sensitivity of 84.2%, and specificity of 96.2%.

**Conclusions and clinical importance:**

Hyperthyroid cats with higher plasma TT4 and lower creatinine concentrations at diagnosis are likely to require >5 mg total daily dose of thiamazole to achieve euthyroidism.

## Introduction

Hyperthyroidism is the most common endocrinopathy in cats in United States, Canada, United Kingdom, Europe, Australia, New Zealand, and Japan, with a prevalence of 6%-21% in senior cats.^1–5^ Anti-thyroid medication, such as thiamazole (also known as methimazole) and carbimazole, is commonly used either as long-term therapy or prior to more definitive treatments (surgery or radioactive iodine).^6^ Thiamazole, a thionamide anti-thyroid agent, inhibits the action of thyroid peroxidase (TPO),^7^ which is an essential enzyme for thyroid hormone synthesis by catalyzing the iodination of tyrosine residues in thyroglobulin and the oxidative coupling of iodinated tyrosines.^8^ The recommended starting dose for thiamazole (Thyronorm® [Norbrook Laboratories Limited, Newry, UK],^9^ Felimazole® [Dechra Limited, Skipton, UK],^10,11^ Thiafeline® [Le Vet Beheer B.V., Oudewater, The Netherlands]) is 5 mg per day, usually given as 2.5 mg tablet or oral solution twice daily.^12,13^ Dose adjustment is often required according to the clinical response (ie, appetite, body weight) and serum total thyroxine (TT4) concentration after the initiation of treatment. Like many other medical conditions, the severity of disease varies across a spectrum. In general, the severity of hyperthyroidism correlates with the degree of elevation in serum TT4 concentration and thyroid gland enlargement.^14^ To date, no definitive guidance on adjusting the starting dose according to the severity of hyperthyroidism at diagnosis is provided by drug manufacturers of methimazole-containing products, although expert panel opinion has suggested that a higher starting dose (ie, a total daily dose of 10 mg) might be considered for cats with severe hyperthyroidism.^13^ Nonetheless, the dose-dependent effect of thiamazole on lowering serum TT4 concentrations in hyperthyroid cats remains largely unexplored.

Chronic kidney disease (CKD) is another common medical condition that affects older cats, with a prevalence of 31%-81% reported in cats over 15 years of age.^15,16^ A wealth of evidence supports the profound influence of thyroid status on renal function. Hyperthyroidism leads to an increased glomerular filtration rate (GFR) because of enhanced cardiac output by positive chronotropic and inotropic effects,^17,18^ reduced peripheral vascular resistance,^19^ increased renal blood flow^20^ and stimulation of the renin-angiotensin-aldosterone system.^21,22^ In human patients with hyperthyroidism, GFR decreases by an average of 29% once euthyroidism was restored.^20^ GFR reduction of 44% and 47% is documented in hyperthyroid cats treated with bilateral thyroidectomy^23^ and thiamazole.^24^ Iatrogenic hypothyroidism further exacerbates renal dysfunction in cats with CKD.^25,26^ Therefore, it is crucial to identify and preferably prevent the development of iatrogenic hypothyroidism, especially in cats with pre-existing CKD or those at increased risk of developing CKD.

Considering the pharmacological mechanism of thiamazole in the inhibition of thyroid hormone synthesis, it is reasonable to assume that hyperthyroid cats with higher plasma TT4 concentration at diagnosis would require a higher dose of thiamazole to restore euthyroidism. Therefore, the aims of this study were first, to develop an algorithm to predict the optimal starting daily dose of thiamazole required to achieve euthyroidism; second, to compare the proportion of cats that developed azotemic CKD between different thiamazole doses; and third, to evaluate whether there was any association between thiamazole dose and survival.

## Materials and methods

### Case selection

Clinical records of the Royal Veterinary College (RVC) Ageing Cat Clinic, which was held in 2 first-opinion practices in London, over a 11-year period (between January 1, 2011 and December 31, 2021) were reviewed and senior cats (≥9 years old) with hyperthyroidism were retrospectively identified for the algorithm training cohort. Clinical records over a subsequent 3-year period (between January 1, 2022 and December 31, 2024) were reviewed to identify a separate testing cohort of cats with hyperthyroidism. All cats examined in these clinics were enrolled as part of an ongoing longitudinal observational study of which informed consent of the owner was obtained and approval of the RVC Ethics and Welfare Committee (URN 2023 2225-3) was granted. A diagnosis of hyperthyroidism was defined as plasma TT4 concentration >55 nmol/L, or TT4 40-55 nmol/L with thyroid stimulating hormone (TSH) < 0.03 ng/mL, together with compatible clinical signs (ie, weight loss and tachycardia in addition to increased appetite or palpation of goiter).

Inclusion criteria for this retrospective study included cats with hyperthyroidism treated with thiamazole (Thyronorm® or Felimazole®). In accordance with our standardized clinic protocol, a starting daily dose of thiamazole of 5 mg and 10 mg was recommended in hyperthyroid cats with a baseline TT4 < 100 nmol/L or ≥ 100 nmol/L, respectively. A follow-up visit was recommended 3-4 weeks after the initiation of thiamazole for dose adjustment. If plasma TT4 concentration was < 10 nmol/L at the follow-up visit but the cat remained clinically well, non-azotemic (plasma creatinine < 2 mg/dL), or had stable CKD (≤25% increase in creatinine compared to the pre-thiamazole value), the same thiamazole dose was maintained and plasma TT4 was re-evaluated after 16 weeks. If the second TT4 is persistently low (<10 nmol/L) then the daily total dose of thiamazole was decreased by approximately 20%-25%. If plasma creatinine increased by >25% from the pre-thiamazole value and plasma TT4 concentration was <10 nmol/L after the initiation of thiamazole, the thiamazole dose was reduced by approximately 20%-25%. Plasma TT4 was re-evaluated 3-4 weeks after dose adjustment. The thiamazole dose at which the cat achieved euthyroidism (plasma TT4 10-40 nmol/L) was recorded and included in the study analysis.

Cats with hyperthyroidism treated with surgical thyroidectomy, radioactive iodine (I131), or transdermal methimazole were excluded. Hyperthyroid cats with no baseline TT4, no follow-up visits, or no information on thiamazole dose when hyperthyroidism was controlled, or cats that took over a year to restore euthyroidism (TT4 10-40 nmol/L) after starting anti-thyroid medication were also excluded.

Cats that developed azotemic CKD within a year after euthyroidism was restored and those that remained non-azotemic for at least a year were identified. Azotemic CKD diagnosis was defined as a plasma creatinine concentration ≥2 mg/dL with a USG < 1.035, or plasma creatinine concentration ≥2 mg/dL on 2 consecutive occasions with no evidence of pre-renal causes including iatrogenic hypothyroidism. Cats that developed azotemic CKD after achieving euthyroidism were classified as International Renal Interest Society (IRIS) stage 2 or 3 according to their plasma creatinine concentration at the time of CKD diagnosis.^27^

### Data collection

Blood samples were collected by jugular venipuncture into heparinized tubes, while urine was obtained by cystocentesis. Samples were stored at 4 °C for <6 h before centrifugation and separation. Biochemical analysis was performed on heparinized plasma at an external laboratory (IDEXX laboratories, Wetherby, UK), TT4 and TSH were measured using chemiluminescence immunoassay, while creatinine was measured using enzymatic colorimetric assay. ﻿In-house urinalyses, including urine specific gravity (USG) measurement by refractometry, dipstick chemistry analysis, and microscopic urine sediment examination, were performed on the day of collection. Doppler method was used to measure systolic blood pressure (SBP).^28^

Clinical records were reviewed to extract: age, sex, breed, body weight, body condition score (BCS, 9-point scale), muscle condition score (MCS, 4-point scale), heart rate, plasma concentrations of creatinine, urea, total calcium, phosphate, potassium, sodium, chloride, total protein, albumin, bilirubin, cholesterol, and TT4 and the activities of alanine aminotransferase (ALT) and alkaline phosphatase (ALP), packed cell volume (PCV), SBP, and USG. Dose of thiamazole and when hyperthyroidism was controlled with plasma TT4 of 10-40 nmol/L for the first time within a year since diagnosis, and the number of dose adjustment required to achieve euthyroidism were recorded and included in the study analysis.

### Statistical analysis

Statistical analyses were performed using R software (Version 4.3.1 GUI 1.79 Big Sur ARM build, ﻿R Foundation for Statistical Computing, Vienna, Austria). Type I error rate was set at 0.05. The normality of continuous variables was assessed using the Shapiro–Wilk test and by visual inspection of Q-Q plots. Most data were not normally distributed in the study and therefore numerical data are presented as median [25th, 75th percentile]. Categorical data are presented as percentages. Controlled dose of thiamazole was categorized into 2 groups: “≤5 mg” or “>5 mg.” Number of dose adjustment to achieve euthyroidism was also categorized into 2 groups: “0” or “≥1.”

#### Baseline clinicopathological variables between groups

Comparison of variables at diagnosis between groups (“≤5 mg” or “>5 mg”) was performed by either independent samples *t-*test or Mann–Whitney *U* test for continuous variables with normal or skewed distributions, respectively. Proportion of categorical outcomes was compared using chi-squared test. Spearman’s correlation was used to evaluate the relationship of total daily dose of thiamazole to achieve euthyroidism with baseline plasma TT4 concentration at diagnosis and the change in plasma TT4 concentration once euthyroidism was restored.

#### Risk factors and predictive algorithm for dose of thiamazole

Binary logistic regression was performed to explore predictors associated with thiamazole dose. For univariable analysis, age, body weight, heart rate, SBP, creatinine, urea, phosphate, total calcium, potassium, sodium, chloride, total protein, albumin, ALT, ALP, and PCV were entered as continuous variables, while sex, BCS and MCS were entered as categorical variables. USG was not entered in this analysis because of the substantial (>40%) missing data. The final model was derived by manual backward elimination, with *P* ≤ .05 denoting significance. Hosmer–Lemeshow test was used to assess the goodness-of-fit of the final model, and variance inflation factor was used to check for presence of co-linearity among the significant risk factors (*P* ≤ .05). The performance of the final multivariable model in prediction was assessed by receiver operating characteristic (ROC) curve analysis. Results are reported as odds ratio (OR; 95% CI).

Predicted probability for each thiamazole dose (“≤5 mg” vs “>5 mg”) for controlling hyperthyroidism for each cat was generated from a fitted multivariable logistic regression model. A predicted probability cutoff of 0.5 was applied for classification. The predictive performance was assessed by the accuracy, sensitivity, specificity, negative predictive value (NPV), and positive predictive value (PPV) based on the comparison between the predicted thiamazole dose group classification and the actual thiamazole dose required to achieve euthyroidism.

#### Validation of predictive model

Clinical records between January 1, 2022 and December 31, 2024 (3-year period) were reviewed to identify a separate testing cohort of cats with hyperthyroidism using identical inclusion and exclusion criteria as mentioned above. The algorithm derived on predicting the optimal thiamazole dose to achieve euthyroidism was applied to the testing cohort. The predictive performance was accessed by the accuracy, sensitivity, specificity, NPV, and PPV.

#### Comparison of proportion of cats developing azotemic CKD between thiamazole dose

Chi-square test was performed to compare proportion of cats developing azotemic CKD within a year and those remained non-azotemic for at least a year since hyperthyroidism was controlled between the different thiamazole dose (“≤5 mg” vs “>5 mg”).

#### Association of thiamazole dose with survival

Date of hyperthyroidism diagnosis was defined as baseline, whereas death of all-cause was the event of interest. Survival times were illustrated with a Kaplan–Meier curve and were compared between groups based on thiamazole dose (“≤5 mg” vs “>5 mg”) using log-rank test. Cox proportional hazard analysis was used to assess the association of survival with thiamazole dose to achieve euthyroidism.

## Results

Over the 11-year period (2011-2021), a total of 419 cats with hyperthyroidism were identified, of which 29 cats were excluded because baseline TT4 (*n* = 4) or thiamazole dose (*n* = 25) was not recorded on our electronic database, or they had no follow-up visit after thiamazole was initiated (*n* = 32). Of the remaining 358 cats, 170 cats were further excluded because hyperthyroidism was treated with thyroidectomy (*n* = 37), transdermal methimazole (*n* = 3) or I131 (*n* = 1), or hyperthyroidism remained uncontrolled for greater than a year after diagnosis (*n* = 129).

In total, 188 hyperthyroid cats met the criteria and were enrolled in the training cohort, with 119 cats (63%) controlled with ≤5 mg (range, 1.25-5 mg) and 69 cats (37%) controlled with >5 mg (range, 7.5-20 mg) total daily dose of thiamazole. Table 1 presents the number of cats controlled on various thiamazole doses. Strong positive correlations were found between thiamazole dose to achieve euthyroidism and baseline plasma TT4 concentration at diagnosis and the change in TT4 once restored to a euthyroid state (both r_s_ = 0.76; *P* < .001). Majority of hyperthyroid cats (67%) were controlled on either 5 mg (*n* = 80) or 10 mg (*n* = 46) of thiamazole daily. Domestic shorthair was the most common breed (*n* = 163), followed by domestic longhair (*n* = 21) and one each of the following breeds: British shorthair, Norwegian Forest, Russian blue, and Siamese mix. The median time taken to achieve euthyroidism since diagnosis was 56 [35, 119] days and 70 [42, 175] days for cats with ≤5 and >5 mg thiamazole, respectively, with no significant difference observed between groups (*P* = .2). The number of dose adjustments required to achieve euthyroidism was significantly different between groups, with higher proportion of cats stabilized on >5 mg requiring at least 1 dose adjustment compared to those controlled on ≤5 mg (39% vs 17%; *P* < .001).

**Table 1:** Number of hyperthyroid cats controlled on various thiamazole doses (*n* = 188).

**Group**	**Total daily dose of thiamazole (mg)**	**Number (*n*)**
**≤5 mg (*n* = 119)**	1.25	1 (1%)
	2.5	32 (27%)
	3.75	6 (5%)
	5	80 (67%)
**>5 mg (*n* = 69)**	7.5	8 (12%)
	10	46 (67%)
	12.5	7 (10%)
	15	3 (4%)
	17.5	1 (1%)
	20	4 (6%)

Baseline clinicopathological variables at diagnosis of hyperthyroidism are summarized in Table 2. Hyperthyroid cats controlled on a >5 mg total daily dose of thiamazole had significantly higher plasma TT4, phosphate, and bilirubin concentrations, higher ALT and ALP activities, higher heart rate and USG, and lower plasma concentrations of creatinine, urea, total protein and total calcium, and were younger than cats that were controlled on a ≤5 mg total daily dose of thiamazole. No other significant differences in baseline clinicopathological variables were identified between groups.

**Table 2:** Descriptive statistics on clinicopathological variables at diagnosis of hyperthyroidism in cats, grouped according to the dosage of thiamazole required to achieve euthyroidism (“≤5 mg” vs. “>5 mg”).

**Variables** (reference interval)	**≤5 mg (*n* = 119)**		**>5 mg (*n* = 69)**		*P*-value
	**Median** **[25th, 75th Percentile]**	*n*	Median [25th, 75th Percentile]	*n*	
**Age (years)**	15.5 [13.7, 17.2]	119	14.1 [12.3, 15.7]	68	**.01**
**BCS (“1–3,” “4–6,” “7–9,” *n* [%])**	43 [37], 68 [58], 6 [5]	117	34 [50] 34 [50], 0 [0]	68	.05
**MCS (“0,” “1,” “2,” “3,” *n* [%])**	7 [6], 40 [34], 56 [48], 13 [11]	116	7 [10], 28 [41], 26 [38], 7 [10]	68	.46
**Weight (kg)**	3.21 [2.92, 4.03]	113	3.4 [2.83, 3.81]	70	.48
**Sex (female neutered, *n* [%])**	71 [60]	119	36 [52]	69	.32
**Heart rate (beats per minute)**	200 [180, 223]	119	220 [200, 240]	68	**<.001**
**Albumin (2.5-4.5 g/dL)**	3.1 [3, 3.3]	101	3.1 [3, 3.2]	67	.14
**ALP (≤ 60 U/L)**	58 [44, 81]	101	127 [86, 187]	67	**<.001**
**ALT (5-60 U/L)**	95 [72, 152]	101	260 [144, 439]	67	**<.001**
**Bilirubin (≤0.3 mg/dL)**	0.09 [0.06, 0.13]	79	0.12 [0.1, 0.18]	67	**<.001**
**Chloride (100-124 mEq/L)**	119 [117, 122]	79	119 [118, 121]	67	.82
**Cholesterol (85-154 mg/dL)**	168 [145, 198]	79	161 [143, 186]	67	.19
**Creatinine (0.23-2 mg/dL)**	1.29 [1.1, 1.6]	101	0.94 [0.85, 1.07]	67	**<.001**
**PCV (30%-45%)**	38 [34, 41]	119	38 [35, 41]	68	.41
**Phosphate (2.79-6.81 mg/dL)**	3.88 [3.51, 4.5]	101	4.33 [3.99, 5.21]	67	**<.001**
**Potassium (3.5-5.5 mEq/L)**	3.8 [3.54, 4.27]	81	3.9 [3.7, 4.2]	67	.41
**SBP (<160 mmHg)**	142 [128, 154]	118	147 [132, 160]	69	.16
**Sodium (145-157 mEq/L)**	155 [153, 157]	79	154 [153, 157]	67	.86
**Total calcium (8.2-11.8 mg/dL)**	9.7 [9.4, 10.1]	101	9.5 [9.2, 9.8]	67	**.01**
**Total protein (6-8 g/dL)**	7.4 [7, 7.8]	101	7.1 [6.8, 7.5]	66	**.02**
**Total thyroxine (10-55 nmol/L)**	66.5 [58.1, 85.7]	119	184 [124, 225]	69	**<.001**
**Urea (7.0-27.7 mg/dL)**	31.7 [24.5, 41.3]	79	26.9 [23, 32.1]	67	**.02**
**USG (≥1.035)**	1.023 [1.017, 1.038]	73	1.033 [1.026, 1.042]	35	**.01**

Significant difference between groups (*P* ≤ .05) are highlighted in bold.

Abbreviations: ALP = alkaline phosphatase; ALT = alanine aminotransferase; BCS = body condition score; MCS = muscle condition score; *n* = number of cats; PCV = packed cell volume; SBP = systolic blood pressure; USG = urine specific gravity.

### Risk factors and predictive algorithm for dose of thiamazole

**Figure 1:**
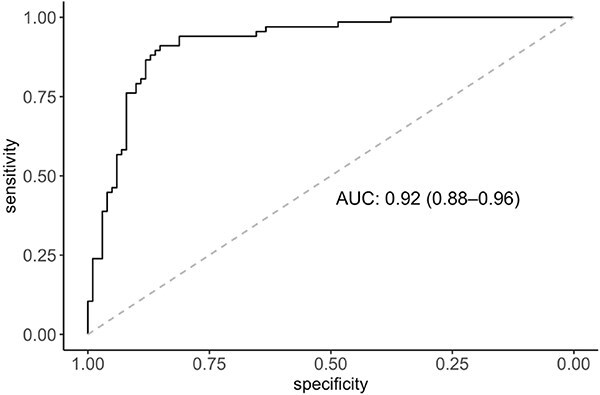
Receiver operating characteristic (ROC) curve illustrating the predictive ability of baseline plasma concentrations of total thyroxine and creatinine at diagnosis of hyperthyroidism for the increased total daily dose (>5 mg) of thiamazole to achieve euthyroidism within a year. Predictive accuracy of this model was excellent with an area under the curve of 0.92 (95% CI, 0.88-0.96).

**Figure 2:**
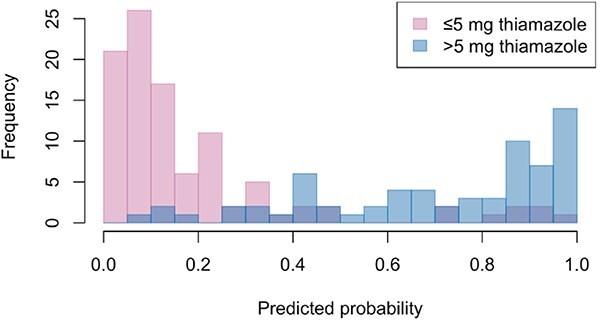
Overlapping histograms illustrating the distribution of predicted probability for cats requiring total daily dose of ≤ 5 mg (pink) and >5 mg (blue) thiamazole to achieve euthyroidism in cats. Predicted probability was generated from the fitted logistic regression model using plasma total thyroxine (TT4) and creatinine concentrations at diagnosis of hyperthyroidism. Areas where the histograms overlap appear as purple.

**Figure 3:**
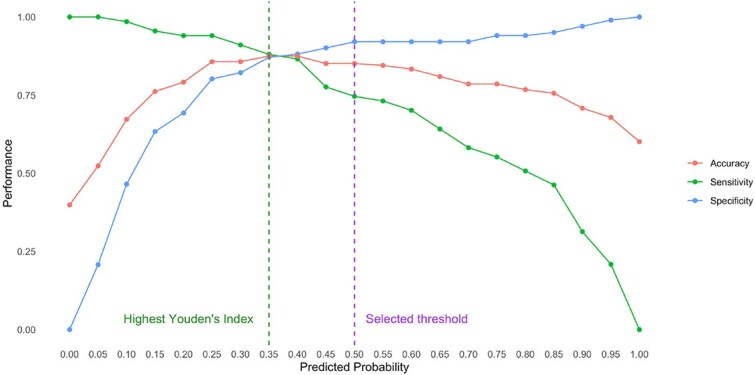
Impacts of predicted probability threshold adjustments on model performance.

Results from univariable logistic regression analysis identified that higher plasma concentrations of TT4, phosphate and bilirubin, ALT and ALP activities, and heart rate, and lower plasma concentrations of creatinine, total protein and total calcium, and being younger at diagnosis were associated with increased thiamazole dose (>5 mg) to achieve euthyroidism (*P* < .1; Table 3). In the final multivariable logistic regression model (*n* = 168), baseline plasma concentrations of TT4 (OR = 1.29 [95%CI, 1.19-1.42] per 10 nmol/L increase; *P* < .001) and decreased creatinine (OR = 0.83 [95%CI, 0.7-0.96] per 0.1 mg/dL increase; *P* = .02) remained significant variables for predicting the thiamazole dose to achieve euthyroidism. The area under the ROC curve was 0.92 (95% CI, 0.88-0.96; Figure 1), suggesting an excellent predictive accuracy of the final multivariable model. Predicted probability for each cat was generated based on the fitted final multivariable logistic regression model (Figure 2). The change in model performance across different predicted probability is illustrated in Figure 3, with the predicted dose (“≤5 mg” or “>5 mg”) for each cat assigned based on probability cutoff of 0.5. The overall accuracy of this predictive model in predicting the optimal dose of thiamazole to achieve euthyroidism was 85.1%, with a sensitivity of 74.6%, specificity of 92.1%, NPV of 84.6%, and PPV of 86.2%.

**Table 3:** Univariable and multivariable logistic regression analysis of factors associated with the higher thiamazole dose (>5 mg) to achieve euthyroidism in cats.

**Variables**	**Univariable analysis**			**Multivariable analysis**		
	OR (95% CI)	*n*	*P*-value	OR (95% CI)	*n*	*P*-value
**Age (year)**	0.87 (0.77-0.96)	187	**.01**			
**Heart rate (bpm × 0.1)**	1.17 (1.07-1.29)	187	**<.001**			
**ALP (U/L × 0.1)**	1.17 (1.1-1.25)	168	**<.001**			
**ALT (U/L × 0.1)**	1.03 (1.02-1.05)	168	**<.001**			
**Bilirubin (mg/dL× 100)**	1.09 (1.04-1.16)	146	**<.001**			
**Creatinine (mg/dL× 10)**	0.68 (0.58-0.77)	168	**<.001**	0.83 (0.7-0.96)	168	**.02**
**Phosphate (mg/dL)**	2.28 (1.52-3.54)	168	**<.001**			
**Total calcium (mg/dL)**	0.41 (0.21-0.76)	168	**.01**			
**Total protein (g/dL × 10)**	0.93 (0.88-0.99)	167	**.02**			
**Total thyroxine (nmol/L × 0.1)**	1.36 (1.26-1.49)	188	**<.001**	1.29 (1.19-1.42)	168	**<.001**
**Urea (mg/dL)**	0.97 (0.94-1)	146	.06			

Significant variables (*P* ≤ .05) in univariable and multivariable models are highlighted in bold.

Abbreviations: ALP = alkaline phosphatase; ALT = alanine aminotransferase; bpm = beats per minute; *n* = number of cats; OR = odds ratio.

### Validation of predictive model

A total of 45 hyperthyroid cats met the inclusion and exclusion criteria and were used as a separate testing cohort for validation. Hyperthyroidism was controlled with ≤5 mg thiamazole in 26 cats (58%) and >5 mg thiamazole in 19 cats (42%). Predicted probability for each cat was generated based on the fitted final multivariable logistic regression model obtained from the training cohort of 168 cats. Using the predicted probability of 0.5 as cutoff, the overall accuracy of this predictive model when applied to a separate testing cohort was 91.1%, with a sensitivity of 84.2%, specificity of 96.2%, NPV of 89.3%, and PPV of 94.1%.

### Development of azotemic CKD after restoration of euthyroidism

Of the 188 hyperthyroid cats enrolled in this study, 23 cats had a diagnosis of azotemic CKD confirmed prior to a diagnosis of hyperthyroidism or before euthyroidism was achieved, 11 cats met the criteria for a diagnosis of azotemic CKD but had suspected iatrogenic hypothyroidism at the same time, 10 cats developed azotemic CKD greater than 1 year after euthyroidism was restored, and 60 cats had insufficient (<1 year) follow-up period once euthyroid to confirm the absence of CKD development. These cats were therefore excluded from further analyses. Of the remaining 84 cats, 22 developed azotemic CKD (IRIS stage 2, *n* = 19; IRIS stage 3, *n* = 3) within a year of euthyroidism being achieved and had no evidence of iatrogenic hypothyroidism, with 14 (63.6%) and 8 (36.4%) cats requiring ≤5 mg and >5 mg total daily dose of thiamazole to restore euthyroidism, respectively (Tables S1 and S2). No difference in plasma creatinine concentration (*P* = .31) or IRIS stage (*P* = 1) at diagnosis of CKD was observed between thiamazole dose groups. Sixty-two cats remained non-azotemic (≤5 mg, *n* = 36 [58.1%]; > 5 mg, *n* = 26 [41.9%]) for a minimum of 1 year after restoration of euthyroidism. There was no difference in the proportion of cats that developed azotemic CKD within a year once euthyroidism was restored between cats controlled on a total daily thiamazole dose of ≤5 and >5 mg (*P* = .65).

### Association of thiamazole dose to achieve euthyroidism with survival

**Figure 4:**
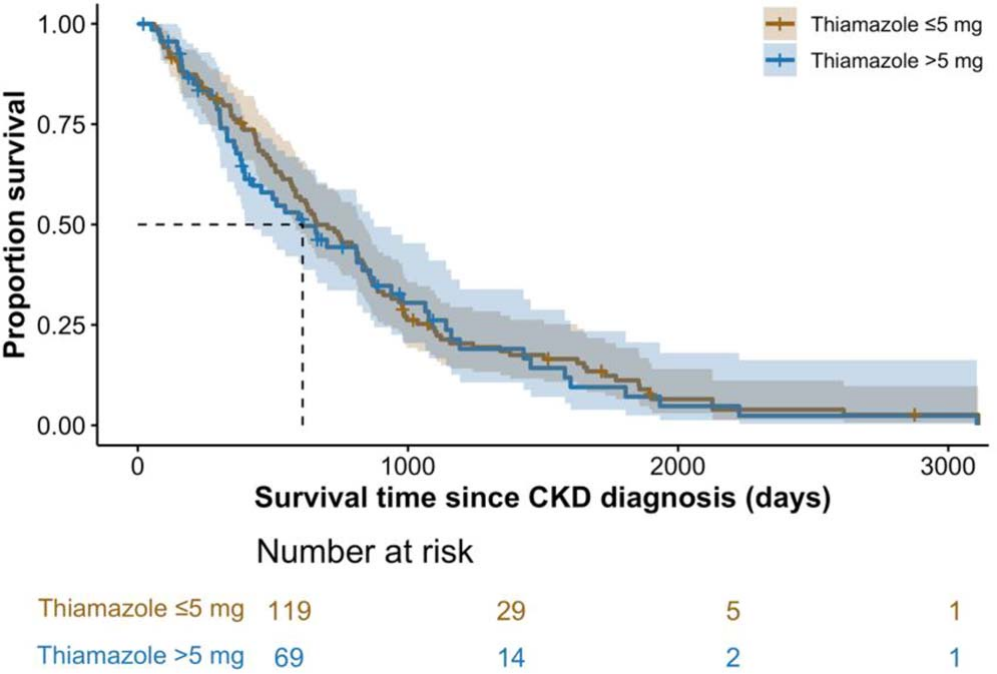
Kaplan–Meier curve illustrating survival in cats with hyperthyroidism (*n* = 188) grouped by the thiamazole dose to achieve euthyroidism (“≤5 mg” vs “>5 mg”). Right censoring was employed for cats that were lost to follow-up or remained alive at the end of study period. No difference in survival time was observed between groups (*P* = .65).

No difference in survival was found between hyperthyroid cats controlled on ≤5 mg thiamazole compared to cats that required >5 mg thiamazole to achieve euthyroidism (*P* = .65), with medium survival time of 661 (95% CI, 584-838) days and 610 (95% CI, 425-874) days, respectively (Figure 4).

## Discussion

The present study provided a reliable algorithm, using plasma concentrations of TT4 and creatinine, to predict the optimal starting daily dose of thiamazole at diagnosis of hyperthyroidism in cats. The predictive model was successfully validated using an additional cohort. The algorithm has the potential to control hyperthyroidism in cats more efficiently, reducing the time taken and the number of veterinary visits and dose adjustment required. Not only would this help to mitigate stress in these hyperthyroid cats, but also alleviate financial burdens associated with recurrent follow-up appointments and repeated plasma TT4 concentration measurements.

Results from our multivariable logistic regression analysis showed that higher plasma TT4 concentration and lower plasma creatinine concentration at hyperthyroidism diagnosis were associated with increased (>5 mg) total daily dose of thiamazole to achieve euthyroidism. It is well-established that thyroxine increases GFR, and muscle mass is often reduced in patients with thyrotoxicosis.^29^ Furthermore, thyroid hormone may also influence the rate of endogenous creatinine production.^30^ Consequently, these factors contribute to the reduced plasma creatinine concentration in hyperthyroid patients with excessive thyroxine production.^23,24,31^ Thiamazole has been suggested to act as a competitive substrate for TPO to decrease the incorporation of iodide into tyrosine molecules for the formation of thyroid hormone.^32,33^ Therefore, a dose-dependent inhibitory effect of thiamazole is expected. This is further supported by our finding that thiamazole dose required to achieve euthyroidism correlates positively with plasma TT4 concentration at diagnosis and the change in TT4 once euthyroid.

An additional explanation for the requirement of the higher dose of thiamazole in cats with higher TT4 and lower creatinine concentrations is that thiamazole is more rapidly cleared by the kidney in the more severely hyperthyroid cats. The route of elimination of thiamazole in the cat has not been studied and while the mean residence time of thiamazole was reported to be shorter in hyperthyroid cats compared to euthyroid (healthy) cats after a single dose,^34^ this difference is unlikely to be clinically significant as it was dependent on 2 of the healthy cats having slow clearance, which increased in these 2 cats on multiple dosing.^35^

Cats controlled on >5 mg thiamazole required higher number of dose adjustments before euthyroidism was achieved, with 39% requiring at least 1 dose adjustment compared to 17% in those controlled on ≤5 mg. Interestingly, if all cats controlled on >5 mg thiamazole had initially been started on the recommended daily dose of 5 mg, at least 1 dose adjustment would certainly be needed regardless. However, 61% of our hyperthyroid cats controlled on >5 mg thiamazole did not require any dose adjustment because our cats with plasma TT4 concentration >100 nmol/L were immediately started on 10 mg total daily dose of thiamazole at diagnosis according to our clinic’s protocol. Therefore, the number of dose adjustments for cats controlled on >5 mg thiamazole reported in our study is likely to be lower than if they had initially started on a total daily dose of 5 mg, due to the additional adjustment required. On the contrary, no significant difference in the time taken to achieve euthyroidism between cats requiring ≤5 and >5 mg thiamazole was observed in our population. There are several explanations for this finding. First, only cats with hyperthyroidism controlled within the first year since diagnosis were included in the study. Second, not all hyperthyroid cats were started on 5 mg total daily dose of thiamazole at diagnosis; those with plasma TT4 > 100 nmol/L at diagnosis were started on 10 mg total daily dose of thiamazole according to our clinic’s protocol. Third, these results could be heavily influenced by the owners, despite all being advised to bring their cats back for the measurement of plasma TT4 concentration 3-4 weeks after each dose adjustment until hyperthyroidism was controlled. However, these factors should not affect the results of our predictive algorithm on the optimal starting dose of thiamazole.

The median survival time for hyperthyroid cats in our study was 610 days, which is longer than the 417 days reported in a previous study of medically treated senior cats (≥9 years old).^36^ However, our study population was highly selected and different to other feline studies.^22,25,36,37^ Notably, only hyperthyroid cats that were successfully restored euthyroidism with thiamazole within a year since diagnosis were included in our analysis. Therefore, the results from our survival analysis could have been overestimated because inadequately treated cats were excluded. While our study was not designed to determine the influence of control of hyperthyroidism on survival, our findings suggested hyperthyroid cats restored to a euthyroid state may have a longer survival than those with either persistent hyperthyroidism or iatrogenic hypothyroidism. Further studies with appropriate survival analysis are needed to confirm these findings. Additionally, survival times and proportion of cats developing azotemic CKD following restoration of euthyroidism were not found to be different between cats that required different dose of thiamazole, providing that euthyroidism was successfully restored.^37^

Overall, we developed an algorithm, using plasma TT4 and creatinine concentrations, to better predict the optimal starting daily dose of thiamazole to achieve euthyroidism in cats. The performance of this predictive model was excellent. The performance of the predictive algorithm was developed and validated using the methodologies for TT4 and creatinine in our clinics; therefore extrapolation to other laboratories or analytical methods, especially those with differing reference intervals, may not be applicable. External validation with a larger data set is warranted to further evaluate the predictive performance of our model. Furthermore, a novel TSH assay based on bulk acoustic wave has recently been developed to measure TSH in cats with enhanced sensitivity and lower limit of quantification.^38^ Future study could explore the potential of incorporating TSH measurement, either using the traditional canine TSH or this novel sensitive assay, to enhance the predictive accuracy of our algorithm in determining the optimal starting dose of thiamazole to achieve euthyroidism.

## Supplementary Material

aalag009_Supplemental_Files

## Data Availability

The data that support the findings of this study are available from the corresponding author upon reasonable request.

## Off-label antimicrobial declaration

Authors declare no off-label use of antimicrobials.

## Institutional animal care and use committee or other approval declaration

Approved by the Ethics and Welfare Committee of the Royal Veterinary College (URN 2023 2225-3).

## Abbreviations

ALP: alkaline phosphatase
ALT: alanine aminotransferase
BCS: body condition score
CKD: chronic kidney disease
GFR: glomerular filtration rate
I131: radioactive iodine
MCS: muscle condition score
MST: medium survival time
NPV: negative predictive value
OR: odds ratio
PPV: positive predictive value
ROC: receiver operating characteristic
SBP: systolic blood pressure
TPO: thyroid peroxidase
TSH: thyroid stimulating hormone
TT4: total thyroxine
USG: urine specific gravity

## References

[1] Wakeling J, Elliott J, Syme H. Evaluation of predictors for the diagnosis of hyperthyroidism in cats. J Vet Intern Med. 2011;25:1057-1065. 21985139 10.1111/j.1939-1676.2011.00790.x

[2] Miyamoto T, Miyata I, Kurobane K, et al. Prevalence of feline hyperthyroidism in Osaka and the Chugoku region. Journal of the Japan Veterinary Medical Association. 2002;55:289-292.

[3] Gójska-Zygner O, Lechowski R, Zygner W. Prevalence of feline hyperthyroidism in mature cats in urban population in Warsaw. Bull Vet Inst Pulawy. 2014;58:267-271.

[4] Bree L, Gallagher BA, Shiel RE, Mooney CT. Prevalence and risk factors for hyperthyroidism in Irish cats from the greater Dublin area. Ir Vet J. 2018;71:2. 10.1186/s13620-017-0113-x PMC5769238 29372047

[5] Köhler I, Ballhausen BD, Stockhaus C, Hartmann K, Wehner A. Prevalence of and risk factors for feline hyperthyroidism among a clinic population in southern Germany. Tierarztliche Praxis Ausgabe K: Kleintiere - Heimtiere. 2016;44:149-157. 10.15654/TPK-150590 26902958

[6] Candellone A, Saettone V, Badino P, et al. Management of feline hyperthyroidism and the need to prevent oxidative stress: what can we learn from human research? Antioxidants. 2021;10:1496. 34573128 10.3390/antiox10091496 PMC8469997

[7] Manna D, Roy G, Mugesh G. Antithyroid drugs and their analogues: synthesis, structure, and mechanism of action. Acc Chem Res. 2013;46:2706-2715. 10.1021/ar4001229 23883148

[8] Roy G, Mugesh G. Bioinorganic chemistry in thyroid gland: effect of antithyroid drugs on peroxidase-catalyzed oxidation and iodination reactions. Bioinorg Chem Appl. 2006;2006:23214. 17497002 10.1155/BCA/2006/23214 PMC1794076

[9] Veterinary Medicines Directorate . Summary of product characteristics - thyronorm. 2021. Accessed June 14, 2024. https://www.vmd.defra.gov.uk/productinformationdatabase/files/SPC_Documents/SPC_997438.PDF

[10] Veterinary Medicines Directorate . Summary of product characteristics - felimazole. 2021; Accessed June 14, 2024. https://www.vmd.defra.gov.uk/productinformationdatabase/files/SPC_Documents/SPC_104872.PDF

[11] Veterinary Medicines Directorate . Summary of product characteristics - thiafeline. 2019; Accessed June 14, 2024. https://www.vmd.defra.gov.uk/productinformationdatabase/files/SPC_Documents/SPC_485702.PDF

[12] Feldman EC, Nelson RW, Reusch CE, Scott-Moncrieff JCR. Canine and Feline Endocrinology. 4th ed. Elsevier Saunders; 2017.

[13] Daminet S, Kooistra HS, Fracassi F, et al. Best practice for the pharmacological management of hyperthyroid cats with antithyroid drugs. J Small Anim Pract. 2014;55:4-13. 10.1111/jsap.12157 24372075

[14] Peterson ME, Broome MR, Rishniw M. Prevalence and degree of thyroid pathology in hyperthyroid cats increases with disease duration: a cross-sectional analysis of 2096 cats referred for radioiodine therapy. J Feline Med Surg. 2016;18:92-103. 10.1177/1098612X15572416 25673019 PMC11149013

[15] Lulich JP, Osborne CA, O’Brien TD, Polzin DJ. Feline renal failure: questions, answers, questions. Compend Contin Educ. 1992;14:127-153.

[16] Marino CL, Lascelles BDX, Vaden SL, Gruen ME, Marks SL. The prevalence and classification of chronic kidney disease in cats randomly selected within four age groups and in cats recruited for degenerative joint disease studies. J Feline Med Surg. 2014;16:465-472. 10.1177/1098612X13511446 24217707 PMC4414065

[17] Hammond HK, White FC, Buxton IL, 0, Saltzstein P, Brunton LL, Longhurst JC. Increased myocardial P-receptors and adrenergic responses in hyperthyroid pigs. Heart Circ Physiol. 1987;252:H283-H290. 10.1152/ajpheart.1987.252.2.H283 3028177

[18] Walker JD, Crawford FA, Kato S, Spinale FG. The novel effects of 3,5,3′-triido-L-thyronine on myocyte contractile function and β-adrenergic responsiveness in dilated cardiomyopathy. J Thorac Cardiovasc Surg. 1994;108:672-679. 7934101

[19] Fazio S, Palmieri EA, Lombardi G, Biondi B. Effects of thyroid hormone on the cardiovascular system. Recent Prog Horm Res. 2004;59:31-50. 10.1210/rp.59.1.31 14749496

[20] Den Hollander JG, Wulkan RW, Mantel MJ, Berghout A. Correlation between severity of thyroid dysfunction and renal function. Clin Endocrinol (Oxf). 2005;62:423-427. 10.1111/j.1365-2265.2005.02236.x 15807872

[21] Asmah B, Wan Nazaimoon W, Norazmi K, Tan T, Khalid B. Plasma renin and aldosterone in thyroid diseases. Horm Metab Res. 1997;29:580-583. 10.1055/s-2007-979105 9479560

[22] Williams TL, Elliott J, Syme HM. Renin-angiotensin-aldosterone system activity in hyperthyroid cats with and without concurrent hypertension. J Vet Intern Med. 2013;27:522-529. 10.1111/jvim.12062 23517505

[23] Graves T, Olivier N, Nachreiner R, Kruger J, Walshaw R, Stickle RL. Changes in renal function associated with treatment of hyperthyroidism in cats. Am J Vet Res. 1994;55:1745-1749. 7887521

[24] Becker T, Graves T, Kruger J, Braselton W, Nachreiner R. Effects of methimazole on renal function in cats with hyperthyroidism. J Am Anim Hosp Assoc. 2000;36:215-223. 10825092 10.5326/15473317-36-3-215

[25] Williams TL, Elliott J, Syme HM. Association of iatrogenic hypothyroidism with azotemia and reduced survival time in cats treated for hyperthyroidism. J Vet Intern Med. 2010;24:1086-1092. 10.1111/j.1939-1676.2010.0566.x 20695989

[26] Williams TL, Elliott J, Syme HM. Effect on renal function of restoration of euthyroidism in hyperthyroid cats with iatrogenic hypothyroidism. J Vet Intern Med. 2014;28:1251-1255. 10.1111/jvim.12359 24773059 PMC4857944

[27] IRIS . Staging of CKD (modified in 2023). https://www.iris-kidney.com/iris-guidelines-1

[28] Syme HM, Barber PJ, Markwell PJ, Elliott J. Prevalence of systolic hypertension in cats with chronic renal failure at initial evaluation. J Am Vet Med Assoc. 2002;220:1799-1804. 10.2460/javma.2002.220.1799 12092951

[29] Nørrelund H, Hove KY, Brems-Dalgaard E, et al. Muscle mass and function in thyrotoxic patients before and during medical treatment. Clin Endocrinol (Oxf). 1999;51:693-699. 10.1046/j.1365-2265.1999.00861.x 10619973

[30] Panciera DL, Lefebvre HP. Effect of experimental hypothyroidism on glomerular filtration rate and plasma creatinine concentration in dogs. J Vet Intern Med. 2009;23:1045-1050. 10.1111/j.1939-1676.2009.0371.x 19678885

[31] Manetti L, Pardini E, Genovesi M, et al. Thyroid function differently affects serum cystatin cand creatinine concentrations. J Endocrinol Invest. 2014;28:346-349. 10.1007/BF03347201 15966508

[32] Jayaram PN, Roy G, Mugesh G. Effect of thione-thiol tautomerism on the inhibition of lactoperoxidase by anti-thyroid drugs and their analogues. J Chem Sci. 2008;120:143-154. 10.1007/s12039-008-0017-0

[33] Papich MG . Saunders Handbook of Veterinary Drugs: Small and Large Animal. 4th ed. Elsevier Saunders; 2016.

[34] Trepanier LA, Peterson ME, Aucoin DP. Pharmacokinetics of methimazole in normal cats and cats with hyperthyroidism. Res Vet Sci. 1991;50:69-74. 2047595 10.1016/0034-5288(91)90055-s

[35] Trepanier LA, Peterson ME, Aucoin DP. Pharmacokinetics of intravenous and oral methimazole following single-and multiple-dose administration in normal cats. J Vet Pharmacol Ther. 1991;14:367-373. 1774813 10.1111/j.1365-2885.1991.tb00850.x

[36] Williams TL, Peak KJ, Brodbelt D, Elliott J, Syme HM. Survival and the development of azotemia after treatment of hyperthyroid cats. J Vet Intern Med. 2010;24:863-869. 10.1111/j.1939-1676.2010.0550.x 20649748

[37] Williams TL, Elliott J, Syme HM. Calcium and phosphate homeostasis in hyperthyroid cats - associations with development of azotaemia and survival time. J Small Anim Pract. 2012;53:561-571. 10.1111/j.1748-5827.2012.01253.x 22860883

[38] Peterson ME, Dougherty E, Rishniw M. Evaluation of a novel, sensitive thyroid-stimulating hormone assay as a diagnostic test for thyroid disease in cats. Am J Vet Res. 2024;85:ajvr.23.12.0278. 10.2460/ajvr.23.12.0278 38382201

