# Supplementary material for: A predictive algorithm for the optimal daily dosage of thiamazole to control cats with hyperthyroidism
Source: J Vet Intern Med. 2026 Feb 3;40(1):aalag009. doi: 10.1093/jvimsj/aalag009 (PMC12866908; doi:10.1093/jvimsj/aalag009)
Supplement: aalag009_Supplemental_Files [file aalag009_supplemental_files.zip › SUPPLEMENTARY_TABLE_1.updated_aalag009.docx]

**SUPPLEMENTARY TABLE 1.** Descriptive statistics on clinicopathological variables at diagnosis of hyperthyroidism in cats, grouped according to development of chronic kidney disease (CKD) within a year after restoration of euthyroidism (“non-CKD” vs. “CKD”) in cats with ≤5 mg total daily dose of thiamazole (n = 50).

| **Variables** (reference interval) | **Non-CKD (n = 36)** | |  | **CKD (n = 14)** | | |  | *P*-value |
| --- | --- | --- | --- | --- | --- | --- | --- | --- |
|  | Median  [25^th^, 75^th^ Percentile] | n |  | Median  [25^th^, 75^th^ Percentile] | n | |  |  |
| Age (years) | 14.5 [12.6, 16.5] | 36 |  | 15.8 [14.4, 16.5] | 14 | |  | 0.29 |
| BCS (“1–3”, “4–6”, “7–9”, n [%]) | 11 [31], 22 [61], 3 [8] | 36 |  | 5 [38], 8 [62], 0 [0] | 13 |  | | 0.68 |
| MCS (“0”, “1”, “2”, “3”, n [%]) | 1 [3], 9 [25], 21 [58], 5 [14] | 36 |  | 1 [8], 7 [54], 4 [31], 1 [8] | 13 |  | | 0.15 |
| Weight (kg) | 3.82 [3.18, 4.47] | 35 |  | 3.13 [2.84, 3.6] | 14 | |  | **0.02** |
| Sex (female neutered, n [%]) | 19 [53] | 36 |  | 9 [64] | 14 | |  | 0.46 |
| Heart rate (beats per minute) | 200 [172, 220] | 36 |  | 198 [180, 235] | 14 | |  | 0.79 |
| Albumin (2.5–4.5 g/dL) | 3.2 [3, 3.3] | 30 |  | 3.1 [3, 3.2] | 13 | |  | 0.28 |
| ALP (≤ 60 U/L) | 67 [46, 85] | 30 |  | 58 [52, 63] | 13 | |  | 0.53 |
| ALT (5–60 U/L) | 108 [78, 152] | 30 |  | 108 [73, 148] | 13 | |  | 0.66 |
| Bilirubin (≤ 0.3 mg/dL) | 0.09 [0.06, 0.16] | 25 |  | 0.07 [0.04, 0.11] | 10 | |  | 0.15 |
| Chloride (100–124 mEq/L) | 120 [118, 122] | 25 |  | 120 [119, 121] | 10 | |  | 0.39 |
| Cholesterol (85–154 mg/dL) | 167 [147, 199] | 25 |  | 182 [156, 203] | 10 | |  | 0.39 |
| Creatinine (0.23–2 mg/dL) | 1.11 [0.91, 1.36] | 30 |  | 1.52 [1.43, 1.69] | 13 | |  | **<0.001** |
| PCV (30–45%) | 40 [36, 42] | 36 |  | 38 [35, 40] | 14 | |  | 0.37 |
| Phosphate (2.79–6.81 mg/dL) | 3.8 [3.52, 4.33] | 30 |  | 3.88 [3.65, 4.39] | 13 | |  | 0.68 |
| Potassium (3.5–5.5 mEq/L) | 3.73 [3.43, 4.03] | 25 |  | 4.1 [3.44, 4.33] | 10 | |  | 0.32 |
| SBP (<160 mmHg) | 145 [131, 156] | 36 |  | 146 [121, 154] | 14 | |  | 0.84 |
| Sodium (145–157 mEq/L) | 155 [154, 156] | 25 |  | 155 [154, 159] | 10 | |  | 0.62 |
| Total calcium (8.2–11.8 mg/dL) | 9.58 [9.41, 9.8] | 30 |  | 10.1 [9.68, 10.36] | 13 | |  | **0.01** |
| Total protein (6–8 g/dL) | 7.4 [6.9, 7.7] | 30 |  | 7.6 [7.3, 7.8] | 13 | |  | 0.31 |
| Total thyroxine (10–55 nmol/L) | 65.7 [58.8, 86.9] | 36 |  | 74.5 [67.1, 88] | 14 | |  | 0.17 |
| Urea (7.0–27.7 mg/dL) | 29.1 [24.1, 34.2] | 25 |  | 42.7 [31.4, 44.1] | 10 | |  | **0.003** |
| USG (≥1.035) | 1.036 [1.023, 1.04] | 18 |  | 1.018 [1.016, 1.03] | 10 | |  | **0.03** |

Significant difference between groups (*P* ≤ 0.05) are highlighted in bold.

Abbreviations: n, number of cats; BCS, body condition score; MCS, muscle condition score; ALP, alkaline phosphatase; ALT, alanine aminotransferase; PCV, packed cell volume; SBP, systolic blood pressure; USG, urine specific gravity.
