# Supplementary material for: A predictive algorithm for the optimal daily dosage of thiamazole to control cats with hyperthyroidism
Source: J Vet Intern Med. 2026 Feb 3;40(1):aalag009. doi: 10.1093/jvimsj/aalag009 (PMC12866908; doi:10.1093/jvimsj/aalag009)
Supplement: aalag009_Supplemental_Files [file aalag009_supplemental_files.zip › SUPPLEMENTARY_TABLE_2.updated_aalag009.docx]

**SUPPLEMENTARY TABLE 2.** Descriptive statistics on clinicopathological variables at diagnosis of hyperthyroidism in cats, grouped according to development of chronic kidney disease (CKD) within a year after restoration of euthyroidism (“non-CKD” vs. “CKD”) in cats with >5 mg total daily dose of thiamazole (n = 34).

| **Variables** (reference interval) | **Non-CKD (n = 26)** | |  | **CKD (n = 8)** | | |  | *P*-value |
| --- | --- | --- | --- | --- | --- | --- | --- | --- |
|  | Median  [25^th^, 75^th^ Percentile] | n |  | Median  [25^th^, 75^th^ Percentile] | n | |  |  |
| Age (years) | 14 [12.8, 15] | 25 |  | 15.8 [14.1, 17.3] | 8 | |  | 0.07 |
| BCS (“1–3”, “4–6”, “7–9”, n [%]) | 12 [46], 14 [54], 0 [0] | 26 |  | 2 [25], 6 [75] | 8 |  | | 0.42 |
| MCS (“0”, “1”, “2”, “3”, n [%]) | 2 [8], 10 [38], 10 [38], 4 [15] | 26 |  | 1 [13], 2 [25], 3 [38], 2 [25] | 8 |  | | 0.83 |
| Weight (kg) | 3.33 [2.94, 3.72] | 26 |  | 3.58 [2.97, 3.69] | 8 | |  | 0.92 |
| Sex (female neutered, n [%]) | 15 [58] | 25 |  | 5 [63] | 8 | |  | 1 |
| Heart rate (beats per minute) | 225 [200, 240] | 26 |  | 221 [208, 252] | 8 | |  | 0.56 |
| Albumin (2.5–4.5 g/dL) | 3 [3, 3.2] | 25 |  | 3.2 [3.1, 3.3] | 8 | |  | 0.12 |
| ALP (≤ 60 U/L) | 106 [76, 209] | 25 |  | 150 [102, 203] | 8 | |  | 0.49 |
| ALT (5–60 U/L) | 294 [105 480] | 25 |  | 261 [205, 426] | 8 | |  | 0.82 |
| Bilirubin (≤ 0.3 mg/dL) | 0.12 [0.09, 0.17] | 25 |  | 0.11 [0.1, 0.17] | 8 | |  | 0.88 |
| Chloride (100–124 mEq/L) | 119 [118, 121] | 25 |  | 119 [119. 121] | 8 | |  | 0.58 |
| Cholesterol (85–154 mg/dL) | 162 [145, 185] | 25 |  | 186 [174, 198] | 8 | |  | 0.21 |
| Creatinine (0.23–2 mg/dL) | 0.97 [0.86, 1.07] | 25 |  | 1.06 [0.96, 1.21] | 8 | |  | 0.12 |
| PCV (30–45%) | 39 [37, 42] | 26 |  | 38 [37, 42] | 8 | |  | 0.67 |
| Phosphate (2.79–6.81 mg/dL) | 4.33 [4.05, 5.47] | 25 |  | 4.62 [4.26, 4.99] | 8 | |  | 0.85 |
| Potassium (3.5–5.5 mEq/L) | 3.9 [3.7, 4.4] | 25 |  | 3.8 [3.5, 4.2] | 8 | |  | 0.49 |
| SBP (<160 mmHg) | 150 [127, 166] | 26 |  | 150 [142, 155] | 8 | |  | 0.9 |
| Sodium (145–157 mEq/L) | 154 [154, 157] | 25 |  | 154 [153, 156] | 8 | |  | 0.4 |
| Total calcium (8.2–11.8 mg/dL) | 9.48 [9.2, 10] | 25 |  | 9.48 [9.41, 9.68] | 8 | |  | 0.75 |
| Total protein (6–8 g/dL) | 7.2 [6.9, 7.6] | 25 |  | 7.2 [6.9, 7.3] | 8 | |  | 0.57 |
| Total thyroxine (10–55 nmol/L) | 165 [124, 217] | 26 |  | 202 [144, 254] | 8 | |  | 0.49 |
| Urea (7.0–27.7 mg/dL) | 25.2 [22.4, 31.7] | 25 |  | 29.4 [27.1, 31.6] | 8 | |  | 0.31 |
| USG (≥1.035) | 1.031 [1.017, 1.04] | 14 |  | 1.04 [1.035, 1.044] | 4 | |  | 0.35 |

Significant difference between groups (*P* ≤ 0.05) are highlighted in bold.

Abbreviations: n, number of cats; BCS, body condition score; MCS, muscle condition score; ALP, alkaline phosphatase; ALT, alanine aminotransferase; PCV, packed cell volume; SBP, systolic blood pressure; USG, urine specific gravity.
